# Dienogest and the Risk of Reoperation in Endometriosis

**DOI:** 10.3390/jpm11090924

**Published:** 2021-09-17

**Authors:** Yong-Soo Seo, Jin-Sung Yuk, Yong-Kyoon Cho, Ji-Yeon Shin

**Affiliations:** 1 Department of Obstetrics and Gynecology, Sanggye Paik Hospital, School of Medicine, Inje University, 1342, Dongil-ro, Nowon-gu, Seoul 01757, Korea; obdrseo@paik.ac.kr (Y.-S.S.); ymkcho@paik.ac.kr (Y.-K.C.); 2 Department of Preventive Medicine, School of Medicine, Kyungpook National University, Daegu 41944, Korea; jyshin@knu.ac.kr

**Keywords:** dienogest, endometriosis, GnRH agonist, reoperation

## Abstract

Background: This retrospective cohort study aimed to determine whether there is a difference in reoperation rates between patients who used dienogest (DNG) and patients who did not use DNG. Methods: Using Health Insurance Review and Assessment Service (HIRA) data generated between 1 January 2010 and 30 June 2018, we identified women with an endometriosis diagnosis code who used GnRH agonists after gynecological surgery. Among them, women prescribed DNG were selected as the DNG group, and those who did not receive DNG were selected as the control group. A survival analysis of the reoperation between the two groups was performed. Results: DNG and control groups were extracted from 9735 people each. The reoperation rates were 0.4% and 0.6% in the DNG and control groups, respectively, without adjusting. In the Cox proportional risk analysis, DNG use increased the reoperation rate {hazard ratio (HR), 1.599; 95% confidence interval (CI), 1.005–2.545}. The site of endometriosis and the number of GnRH agonist injections were not associated with reoperation (HR, 1.008; 95% CI, 0.739–1.374; HR, 1.062; 95% CI, 0.690–1.635). In the subgroup survival analysis, according to the period between the last GnRH agonist injection and the first DNG dose, DNG did not increase the reoperation rates up to 9 months (~3 months: HR, 0.968; 95% CI, 0.551–1.699; 4~6 months: HR, 1.094; 95% CI, 0.58–2.063; 7~9 months: HR, 2.419; 95% CI, 0.735–7.962), but DNG increased the reoperation rate from 10 months onwards (10~12 months: HR, 3.826; 95% CI, 1.164–12.579 and ~13 months: HR, 8.436; 95% CI, 4.722–15.072). Conclusions: Women who used DNG had a higher endometriosis reoperation rate than women who did not use DNG. However, the initiation of DNG treatment within nine months after the last GnRH agonist injection did not affect the endometriosis reoperation rate.

## 1. Introduction

Endometriosis is an estrogen-dependent chronic inflammatory disease in which endometrial glands and stroma exist outside the uterine cavity, causing dysmenorrhea, pelvic pain, infertility, and an ovarian mass [1,2] The incidence of endometriosis is 0.1–0.2% in women of childbearing age, and the prevalence is 1–15.2% [1,3,4,5,6,7,8].

Risk factors for endometriosis include low parity, short breastfeeding duration, short menstrual cycles, early menarche, late menopause, alcohol, low body mass index, and nickel allergy [1,9,10]. The most accepted theory of endometriosis pathogenesis is the retrograde menstruation theory [1,9], which indicates that endometriosis occurs when menstrual blood flows through the fallopian tubes into the abdominal cavity [1,9].

The main purpose of medical treatment for endometriosis is to relieve pain and prevent reoperation. For this purpose, nonsteroidal anti-inflammatory drugs (NSAIDs), gonadotropin-releasing hormone (GnRH) agonists, oral contraceptives (OCs), and oral progestin are mainly used [1,2]. Although GnRH agonists have similar or better effects than OCs or oral progestin for relieving pain in endometriosis, it is recommended that they are used for no more than six months since they cause menopausal symptoms and bone loss with long-term use [2,11,12].

Endometriosis is a common recurrent chronic disease, and the recurrence rate of its symptoms is quite high at 21.5% after two years and 40–50% after five years [2,13]. Koshiba et al. and Adachi et al. reported that the recurrence rate of endometrioma in women treated with dienogest (DNG) was lower than in women not treated with DNG [14,15]. In addition, Lee et al. reported that the recurrence rate of endometrioma in the DNG group was 1.8%, which is lower than that of other treatments [16].

DNG is the progestin used in combination with other estrogens as OCs or for menopausal hormone therapy (MHT) [17]. Since DNG has a strong progestational effect, DNG alone has recently been used as a treatment for endometriosis [17]. However, although the main purpose of medical treatment for endometriosis is to prevent reoperation, few studies have assessed the reoperation rate of patients treated with DNG [1,2].

Therefore, the aim of this retrospective cohort study was to determine whether there is a difference in the reoperation rate between patients who used DNG and patients who did not use it.

## 2. Methods

### 2.1. Study Data

In South Korea, more than 98% of Koreans are obliged to join the national medical insurance system provided by the Korea Health Insurance Corporation [18]. Thus, Korean health insurance data form a kind of cohort for Koreans. These data contain most of the information about the treatment of patients including age, gender, region, insurance characteristics, diagnosis, surgical procedure, prescription drug, dosage, and duration of prescription [18]. The Health Insurance Review and Assessment Service (HIRA) is a public agency that assesses the adequacy of the costs charged by medical institutions and orders them to pay the Korea Health Insurance Corporation [18]. Therefore, HIRA shares most of the medical information owned by the Korea Health Insurance Corporation [18]. This retrospective cohort study used HIRA data from 1 January 2010 to 30 June 2018.

### 2.2. Selection of Participants

We used the International Statistical Classification of Diseases and Related Health Problems 10th edition (ICD-10; diagnosis code), the HIRA Drug Ingredients Codes (drug code) and Health Insurance Medical Care Expenses (2016, 2018 version; surgery code) for patient selection. From 1 January 2010 to 31 December 2017, the data of women with an endometriosis diagnostic code (N80.x) who received a GnRH agonist {167202BIJ (goserelin acetate), 167201BIJ (goserelin acetate), 198501CSI (nafarelin), 182602BIJ (leuprolide acetate), 182604BIJ (leuprolide acetate), 244902BIJ (triptorelin acetate)} at the same time were extracted. Only women who had undergone gynecological surgeries {subserosal myomectomy (R4121), complex myomectomy (R4122), pelvic adhesiolysis (R4160), pelviscopic fulguration (R4165), pelviscopic foreign body removal (R4166), metroplasty of uterine anomaly (R4170), unilateral adnexectomy (R4331), bilateral adnexectomy (R4332), tubaligation with pelviscopy (R4341), tubaligation with pelviscopy (R4342), tubaligation with pelviscopy (R4343), tubaligation with pelviscopy (R4345), salpingostomy or salpingoplasty (R4400), fimbrioplasty (R4405), tubotubal anastomosis (R4411), transposition of ovary (R4413), benign extirpation of adnexal tumor (R4421), ovarian wedge resection (R4430), incision and drainage of ovarian cyst (R4435), tubal or ovarian pregnancy (R4531), cornual pregnancy (R4532), cervical pregnancy (R4533)} for abdominal cavity confirmation within 180 days prior to injection of the first GnRH agonist were extracted. Among them, the patients who were prescribed DNG for 28 days or longer after GnRH agonist administration were selected as the DNG group, and those who did not receive DNG were selected as the control group.

Women who had malignant tumors (Cxx.x) or menopause diseases (N95.x, M81.0, M80.0, E28.3) in the diagnostic code from 1 January 2010 to 30 June 2018, were excluded from the analysis. Women with a hysterectomy procedural code (R4130, 4143~4146, 4154~4155, 4183, 4202, 4203, 4221, 4223, 4250, 4427~4428, 4482, 4507~4510, or 5001~5002) within 180 days after the last GnRH agonist or before the first DNG use were excluded. Women whose last GnRH agonist was before 1 January 2013, were excluded, since the South Korean launch date of DNG was March 2013. The control group was selected for the DNG group using 1: 1 propensity score matching (PSM) according to age at intervals of 5 years, socioeconomic status (SES), number of GnRH agonists, Charlson comorbidity index (CCI) and site of endometriosis.

Women who had an endometriosis diagnostic code (N80.x) as the first or second diagnosis and who underwent laparoscopic or laparotomic gynecological surgery {benign extirpation of adnexal tumor (R4421), bilateral adnexectomy (R4332), fulguration (R4165), total hysterectomy (R4183), incision and drainage of ovarian cyst (R4435), laparotomy (R4345), myomectomy (R4122), ovarian wedge resection (R4430), pelvic adhesiolysis (R4160), surgical fulguration of oviduct (R4342), and unilateral adnexectomy (R4331)} were defined as women who underwent endometriosis reoperation. The starting date for the survival analysis was defined as the day of the last GnRH agonist injection, and the day of the event was defined as the day of reoperation. If no reoperation was performed, the date of the event was defined as the last day of the data (30 June 2018).

### 2.3. Statistics

For the statistical analysis of the PSM and Cox proportional hazards model, each independent variable was defined as follows. The age category was defined as the five-year interval, and if the type of medical insurance was Medicaid, it was defined as low SES. The frequency of GnRH agonist administration was divided into 3 times or fewer, 4–6 times, 7–9 times, 10–12 times, and 13 times or more. If DNG was prescribed for more than 28 days, the patient was defined as using DNG. The duration of DNG prescription was divided into 6 months or fewer, 7–12 months, 13–18 months, 19–24 months, and 25 months or more. The duration between the last GnRH agonist injection and the first DNG dose was divided into 3 months or fewer, 4–6 months, 7–9 months, 10–12 months, and 13 months or more. The site of endometriosis was divided into ovarian endometriosis (N80.1), uterine adenomyosis (N80.0) and other endometrioses (N80.2~N80.9). To correct for the comorbidities, the CCI was calculated for the period from the day of the last GnRH agonist injection to the day before 365 days, as described by Quan et al. [19].

All statistical analyses were performed using SAS enterprise guide 6.1 (SAS Institute Inc., Cary, NC, USA), and survival analysis plots were drawn using R 3.0.2 (The R Foundation for Statistical Computing, Vienna, Austria). All statistical analyses were performed by a two-sided test and defined as statistically significant when the *p*-value was less than 0.05. A *t*-test and Mann-Whitney U test were used to analyze the continuous variables, and the chi-square test and Fisher’s exact test were used to analyze the categorical variables. The Cochran-Armitage trend test was used to identify trends in the order of categorical variables. The log-rank test was used for the survival analysis of binary variables, and the Cox proportional hazards model was used to calculate the influence of various variables.

### 2.4. Ethics

This study was approved by the Eulji Hospital Institutional Review Board (IRB; EMCS-2019-01-007). Since this study used retrospective data and deidentified variables to identify individuals, this study cannot do any harm to any individual. Thus, informed consent was not required from patients according to the Bioethics and Safety Act in South Korea. Although this study used data provided by HIRA, HIRA had no impact on the results of this study.

## 3. Results

**Figure 1:**
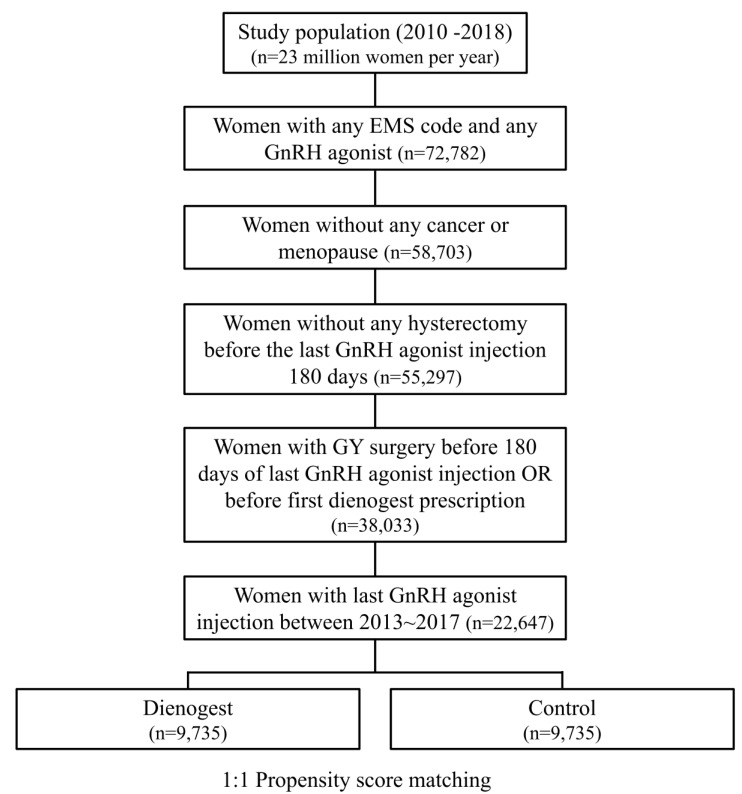
Flowchart to select cases and controls according to DNG use in the HIRA 2010–2018 data. GnRH, gonadotropin-releasing hormone; GY, gynecology; HIRA, The Health Insurance Review and Assessment Service.

From the total HIRA dataset, the data of a total of 22,647 women were extracted, including 10,379 women using DNG and 12,268 women not using DNG (Figure 1). The average age of all patients was 32.73 ± 0.04 years. Table 1 shows the yearly characteristics of these women. PSM was performed on the extracted data, and DNG and control groups were established from 9735 people per condition (Figure 1). The mean age was 32.46 ± 0.07 years and 32.70 ± 0.07 years (*p*-value < 0.001), and the reoperation rate was 0.4% and 0.6% in the DNG group and the control group, respectively, without adjusting (Table 2). The mean GnRH agonist injection count was 4.05 ± 0.01 in the DNG group and 4.08 ± 0.02 in the control group (*p*-value 0.1). The mean duration of DNG use in the DNG group was 12.55 ± 0.10 months, and the mean duration from the last GnRH agonist injection to the first DNG dose was 100.96 ± 2.06 days. The detailed characteristics of the patients are shown in Table 2.

In the log-rank test for reoperation, the reoperation rate of the DNG group was higher than that of the control group (*p*-value 0.047). In the Cox proportional risk analysis, which was adjusted for age, SES, and CCI, the reoperation was increased in the DNG group compared to the control group {hazard ratio (HR), 1.599; 95% confidence interval (CI), 1.005–2.545} (Table 3). The site of endometriosis and the number of GnRH agonist injections were not associated with reoperation (HR, 1.008; 95% CI, 0.739–1.374 and HR, 1.062; 95% CI, 0.690–1.635). In the Cox proportional risk analysis of patients in the DNG group, the reoperation rate increased as the period between the last GnRH agonist injection and the first DNG dose increased (HR, 1.809; 95% CI, 1.507–2.171). In other words, the reoperation rate was higher in patients in whom DNG was prescribed later rather than sooner. However, the duration of DNG, the site of endometriosis and the number of GnRH agonist injections were not associated with reoperation (HR, 1.063; 95% CI, 0.854–1.321; HR, 1.142; 95% CI, 0.785–1.660; and HR, 0.911; 95% CI, 0.507–1.639; Table 3).

In the subgroup survival analysis based on the period between the last GnRH agonist injection and the first DNG dose, DNG treatment did not increase the reoperation rate until 9 months (~3 months: HR, 0.968; 95% CI, 0.551–1.699; 4~6 months: HR, 1.094; 95% CI, 0.58–2.063; and 7~9 months: HR, 2.419; 95% CI, 0.735–7.962) but DNG increased the reoperation rate from 10 months onward (10~12 months: HR, 3.826; 95% CI, 1.164–12.579 and ~13 months: HR, 8.436; 95% CI, 4.722–15.072; Table 4).

## 4. Discussion

In our study, patients treated with DNG had a higher reoperation rate of endometriosis than patients in the control group. These results are different from those of previous studies [14,15,16]. Adachi et al. and Koshiba et al. reported that compared to placebo, DNG reduces the recurrence of endometrioma [14,15,16]. Lee et al. reported that the endometrioma recurrence rate of DNG was 1.8%, which was lower than that of other treatments (such as a levonorgestrel-releasing intrauterine system or OCs) in other studies [16]. The reasons for the differences from previous studies can be interpreted as follows. First, previous studies confirmed the recurrence of endometrioma using ultrasound [14,15,16]. However, in this study, target diseases included all types of endometrioses, as well as endometrioma, and recurrence was defined as reoperation, not a positive ultrasound result. Second, unlike previous studies, this study targeted only patients with endometriosis who used GnRH agonists after gynecologic surgery. Previous studies did not have a control group or used a postoperative GnRH agonist [14,15,16]. Third, unlike the previous studies, this study also included women who used DNG after a certain period of time after the last GnRH agonist injection [14,15,16]. Fourth, the follow-up period in this study was up to six years, unlike other studies (up to two years) [14,15,16]. Additionally, this study had a different research design than previous studies. Therefore, care must be taken when interpreting this the results of this study in comparison with those of previous studies.

However, it is difficult to assert that DNG increases the reoperation rate of endometriosis since beginning DNG treatment within nine months after the last GnRH agonist injection did not affect the reoperation rate of endometriosis. Risk factors for endometriosis include low parity, short menstrual cycles, early menarche, and late menopause [1,9]. These risk factors share a common feature in that they increase ovulation and menstruation throughout the lifetime [1]. Therefore, to reduce endometriosis recurrence, the number of ovulations or menstruation cycles should be reduced [1]. However, DNG inhibits ovulation and, in many cases, hypomenorrhea or amenorrhea [16,17,20]. Therefore, it would be reasonable to identify factors other than DNG itself that increase the recurrence rate of endometriosis. Menstruation resumes three to four months after discontinuation of the GnRH agonist [21]. Thus, patients in our cohort had no menstruation cycle within three months and had five or six menstruation cycles within nine months of the last GnRH agonist injection. The longer the period between the last GnRH agonist injection and the first DNG dose, the greater the number of ovulation and menstruation cycles becomes. Our results showed that patients using DNG within nine months after the last GnRH agonist had a lower reoperation rate than patients using DNG after nine months (Table 4). The shorter the period is, the lower the reoperation rate (Table 4). Therefore, the use of DNG before menstruation resumes following the last GnRH agonist injection is an important factor in reducing the reoperation rate of endometriosis.

The duration of DNG usage and the reoperation rate were not related in our results. If the duration of DNG is related to the reoperation rate of endometriosis, the prolonged use of DNG would lower the reoperation rate because it reduces the chance of ovulation and retrograde menstruation [16,17,20]. However, our results showed that the duration of DNG use was not related to the reoperation rate (Table 3).

This is likely due to a mechanism other than menstrual frequency affecting the reoperation rate. The exact mechanism affecting the reoperation rate is unknown. However, it should be noted that the study only included patients who used postoperative GnRH agonists. In addition, this result together with the initiation time of DNG after the last GnRH agonist injection indicates that there may be a critical treatment window for the initiation of DNG.

To clarify this point, we searched for the initiation time of progestin or OC treatment after GnRH agonist treatment, but no relevant studies were found. Further studies on the duration and initiation time of DNG after GnRH agonist treatment are needed.

The number of GnRH agonist injections was not related to the reoperation rate of endometriosis. Most patients used GnRH agonists within three months, with an average GnRH agonist injection rate of 4.05 ± 0.01. Previous studies on this topic have shown inconsistent results. One meta-analysis reported that GnRH agonist use for six months produced a lower recurrence rate of endometriosis than GnRH agonist use for three months [22]. However, this study was not a direct meta-analysis comparing GnRH agonist for six months and GnRH agonist for three months, but an indirect comparison network meta-analysis. In contrast, a randomized trial comparing nafarelin use for six months and nafarelin use for three months reported no difference in symptom improvement and recurrence rates [23]. Since we did not analyze GnRH agonists by type, care must be given when interpreting our results.

Our study has some limitations. First, the follow-up regularity may have affected the results. Patients who were prescribed DNG may have followed a more regular follow-up schedule than patients not prescribed DNG. More frequent follow-ups may have led to reoperation through the early detection of endometriosis, such as endometrioma, using ultrasound. However, our study did not account for this, and further study may be required. Second, the follow-up period of this study was up to six years. Endometriosis is a common recurrent chronic disease that continues until menopause. However, because the mean age of the patients in this study was 32.58 ± 0.05 years, a longer period of study is needed. Third, this study could not identify the stage of endometriosis, parity, body mass index, detailed surgical information and the usage of oral contraceptive drug due to the characteristics of the insurance data. Therefore, these could not be statistically corrected.

## 5. Conclusions

Women who used DNG had a higher reoperation rate of endometriosis than women who did not use DNG. Specially, the reoperation was statistically more prevalent in patients who were given DNG later than ten months after the last GnRH agonist injection. However, the initiation of DNG treatment within nine months after the last GnRH agonist injection did not affect the reoperation rate of endometriosis.

## Figures and Tables

**Table 1:** Characteristics of the women with EMS injected with GnRH agonist according to the year in the HIRA 2013–2018 data.

	2013	2014	2015	2016	2017	Total	*p*-Value
	Number (%)/Mean ± SE						
Number of women with EMS	4669 (100%)	4612 (100%)	4585 (100%)	4592 (100%)	4887 (100%)	22,647 (100%)	<0.001
Dienogest	1503 (32.5%)	2054 (45.5%)	2113 (47.6%)	2220 (50.3%)	2489 (53.6%)	10,379 (45.8%)	
No dienogest	3128 (67.5%)	2461 (54.5%)	2324 (52.4%)	2196 (49.7%)	2159 (46.5%)	12,268 (54.2%)	
Mean age of women with EMS (years)	32.38 ± 0.10	32.59 ± 0.10	32.58 ± 0.10	33.08 ± 0.10	33.04 ± 0.10	32.73 ± 0.04	<0.001 ^a^
SES							0.488
Mid~high SES	4593 (99.2%)	4483 (99.3%)	4403 (99.2%)	4384 (99.3%)	4601 (99.0%)	22,464 (99.2%)	
Low SES	38 (0.8%)	32 (0.7%)	34 (0.8%)	32 (0.7%)	47 (1.0%)	183 (0.8%)	

Data are expressed as the number (%) or mean ± standard error. EMS, endometriosis; GnRH, gonadotropin-releasing hormone; HIRA, The Health Insurance Review and Assessment Service; SE, standard error; SES, socioeconomic status. ^a^ Mann-Whitney U test.

**Table 2:** Characteristics of the women with EMS injected with a GnRH agonist after PSM in the HIRA 2013–2018 data.

	No Dienogest (n = 9735)	Dienogest (n = 9735)	Total (n = 19,470)	*p*-Value
Mean age of women (years)	32.70 ± 0.07	32.46 ± 0.07	32.58 ± 0.05	<0.001 ^a^
SES				0.476
Mid~high SES	9650 (99.1%)	9659 (99.2%)	19,309 (99.2%)	
Low SES	85 (0.9%)	76 (0.8%)	161 (0.8%)	
CCI				0.126
0	7896 (40.6%)	7935 (40.8%)	15,831 (81.3%)	
1	1153 (5.9%)	1177 (6.1%)	2330 (12.0%)	
2	585 (3.0%)	514 (2.6%)	1099 (5.6%)	
3	92 (0.5%)	105 (0.5%)	197 (1%)	
4	7 (0.0%)	4 (0.0%)	11 (0.1%)	
5	0 (0.0%)	0 (0.0%)	0 (0.0%)	
6	2 (0.0%)	0 (0.0%)	2 (0.0%)	
Diagnosis code				<0.001
N80.0 (Uterus)	745 (7.7%)	588 (6.0%)	1333 (100%)	
N80.1 (Ovary)	7493 (77.0%)	7615 (78.2%)	15,108 (100%)	
N80.2 (Fallopian tube)	32 (0.3%)	20 (0.2%)	52 (100%)	
N80.3 (Pelvic peritoneum)	540 (5.6%)	592 (6.1%)	1132 (100%)	
N80.4 (Rectovaginal septum)	15 (0.2%)	14 (0.1%)	29 (100%)	
N80.0 (Intestine)	8 (0.1%)	7 (0.1%)	15 (100%)	
N80.0 (Cutaneous scar)	1 (0%)	1 (0%)	2 (100%)	
N80.0 (Other)	181 (1.9%)	88 (0.9%)	269 (100%)	
N80.0 (Unspecified)	720 (7.4%)	810 (8.3%)	1530 (100%)	
Number of GnRH agonist injections				0.065
1~3	4590 (47.2%)	4594 (47.2%)	9184 (100%)	
4~6	5018 (51.6%)	4983 (51.1%)	10,001 (100%)	
7~9	93 (1.0%)	97 (1.0%)	190 (100%)	
10~12	31 (0.3%)	51 (0.5%)	82 (100%)	
12~	3 (0.0%)	10 (0.1%)	13 (100%)	
Duration of dienogest (months)				
1~6		3238 (33.3%)	3238 (33.3%)	
7~12		2677 (27.5%)	2677 (27.5%)	
13~18		1903 (19.6%)	1903 (19.6%)	
19~24		891 (9.2%)	891 (9.2%)	
25~		1026 (10.6%)	1026 (10.6%)	
Duration between last GnRH agonist injection and first dienogest treatment (months)				
1~3		7225 (74.2%)	7225 (74.2%)	
4~6		1254 (12.9%)	1254 (12.9%)	
7~9		403 (4.1%)	403 (4.1%)	
10~12		225 (2.3%)	225 (2.3%)	
12~		628 (6.5%)	628 (6.5%)	
Reoperation of women with EMS				
Total				0.291
No reoperation	9703 (99.7%)	9694 (99.6%)	19,397 (100%)	
Reoperation	32 (0.3%)	41 (0.4%)	73 (100%)	
EMS of the uterus				0.312 ^b^
No reoperation	742 (99.6%)	583 (99.2%)	1325 (100%)	
Reoperation	3 (0.4%)	5 (0.9%)	8 (100%)	
EMS of the ovaries				0.94
No reoperation	7466 (99.6%)	7587 (99.6%)	15,053 (100%)	
Reoperation	27 (0.4%)	28 (0.4%)	55 (100%)	
Operation-free time (day)				
Total	2000 ± 1.0	1996 ± 1.4		
Ovarian EMS	1999 ± 1.2	1997 ± 1.5		
Uterine EMS	1997 ± 4.0	1985 ± 8.3		
Other EMS	2003 ± 1.6	1995 ± 3.5		

Data are expressed as the number (%) or mean ± standard error. CCI, Charlson comorbidity index; EMS, endometriosis; GnRH, gonadotropin-releasing hormone; HIRA, The Health Insurance Review and Assessment Service; SE, standard error; SES, socioeconomic status. ^a^ Mann-Whitney U test. ^b^ Fisher’s exact test.

**Table 3:** Cox proportional hazard model for reoperation according to dienogest start time in the HIRA 2013–2018 data.

Dienogest and Control			Only Dienogest		
	HR (95% CI)	*p*-Value		HR (95% CI)	*p*-Value
Unadjusted HR			Unadjusted HR		
Age per 5 years	1.289 (1.085–1.531)	0.004	Age per 5 years	1.272 (1.013–1.596)	0.038
SES	0 (0-Infinite)	0.995	SES	0 (0-Infinite)	0.996
Dienogest	1.594 (1.002–2.537)	0.049	Duration between last GnRH agonist injection and first dienogest ^a^	1.767 (1.48–2.11)	<0.001
Number of GnRH agonist injections ^a^	0.996 (0.648–1.532)	0.986	Number of GnRH agonist injections ^a^	1.107 (0.634–1.933)	0.721
CCI	1.07 (0.756–1.515)	0.702	CCI	1.284 (0.853–1.932)	0.23
Site of EMS ^b^	1.007 (0.739–1.371)	0.965	Site of EMS ^b^	1.22 (0.845–1.761)	0.289
			Duration of dienogest ^a^	0.963 (0.772–1.202)	0.739
Adjusted HR formula 1			Adjusted HR formula 1		
Age per 5 years	1.288 (1.084–1.531)	0.004	Age per 5 years	1.338 (1.06–1.69)	0.014
SES	0 (0-Infinite)	0.995	SES	0 (0-Infinite)	0.996
Dienogest	1.601 (1.006–2.547)	0.047	Duration between last GnRH agonist injection and first dienogest treatment ^a^	1.8 (1.506–2.152)	<0.001
Adjusted HR formula 2			Adjusted HR formula 2		
Age per 5 years	1.291 (1.086–1.535)	0.004	Age per 5 years	1.338 (1.057–1.693)	0.016
SES	0 (0-Infinite)	0.995	SES	0 (0-Infinite)	0.996
Dienogest	1.599 (1.005–2.545)	0.048	Duration between last GnRH agonist injection and first dienogest treatment ^a^	1.809 (1.507–2.171)	<0.001
Number of GnRH agonist injections ^a^	1.062 (0.690–1.635)	0.785	Number of GnRH agonist injections ^a^	0.911 (0.507–1.639)	0.756
CCI	1.064 (0.751–1.507)	0.728	CCI	1.259 (0.833–1.903)	0.274
Site of EMS ^b^	1.008 (0.739–1.374)	0.961	Site of EMS ^b^	1.142 (0.785–1.660)	0.488
			Duration of dienogest treatment ^a^	1.063 (0.854–1.321)	0.586

CCI, Charlson comorbidity index; CI, confidence interval; EMS, endometriosis; GnRH, gonadotropin-releasing hormone; HIRA, The Health Insurance Review and Assessment Service; HR, hazard ratio; SES, socioeconomic status. ^a^ The category of this variable is as shown in Table 2 (GnRH agonist; 1~3, 4~6. 7~9, 10~12, and 12~. Dienogest initiation; 1~3, 4~6, 7~9, 10~12, and 12 months~. Dienogest duration; 1~6, 7~12, 13~18, 19~24, and 25 months~). ^b^ The category of this variable is as shown in Table 3 (ovarian EMS, uterine EMS, other EMS).

**Table 4:** Cox proportional hazard model for the reoperation rate according to dienogest initiation time in the HIRA 2013–2018 data.

	Total		~3 Months		4~6 Months		7~9 Months		10~12 Months		13 Months~	
	HR (95% CI)	*p*-Value	HR (95% CI)	*p*-Value	HR (95% CI)	*p*-Value	HR (95% CI)	*p*-Value	HR (95% CI)	*p*-Value	HR (95% CI)	*p*-Value
Unadjusted HR												
Age per 5 years	1.289 (1.085–1.531)	0.004	1.297 (1.041–1.617)	0.021	1.343 (1.043–1.728)	0.022	1.263 (0.984–1.622)	0.067	1.388 (1.076–1.791)	0.012	1.265 (1.028–1.557)	0.026
SES	0 (0-Infinite)	0.995	0 (0-Infinite)	0.996	0 (0-Infinite)	0.996	0 (0-Infinite)	0.996	0 (0-Infinite)	0.996	0 (0-Infinite)	0.996
Dienogest	1.594 (1.002–2.537)	0.049	0.707 (0.370–1.35)	0.293	0.82 (0.251–2.677)	0.742	2.287 (0.700–7.468)	0.171	3.625 (1.11–11.84)	0.033	7.064 (4–12.48)	<0.001
Number of GnRH agonist injections ^a^	0.996 (0.648–1.532)	0.986	0.918 (0.527–1.602)	0.764	1.026 (0.549–1.918)	0.935	0.937 (0.498–1.765)	0.841	0.858 (0.454–1.622)	0.638	0.749 (0.44–1.275)	0.287
CCI	1.07 (0.756–1.515)	0.702	0.926 (0.565–1.517)	0.759	0.962 (0.558–1.658)	0.888	0.72 (0.366–1.418)	0.342	0.717 (0.364–1.413)	0.336	0.993 (0.642–1.537)	0.976
Site of EMS ^b^	1.007 (0.739–1.371)	0.965	0.927 (0.611–1.406)	0.72	0.827 (0.497–1.373)	0.462	0.769 (0.452–1.311)	0.335	0.766 (0.45–1.307)	0.328	0.76 (0.488–1.184)	0.224
Adjusted HR formula 1												
Age per 5 years	1.288 (1.084–1.531)	0.004	1.299 (1.041–1.621)	0.021	1.343 (1.043–1.729)	0.022	1.268 (0.987–1.628)	0.063	1.392 (1.077–1.797)	0.011	1.313 (1.063–1.62)	0.011
SES	0 (0-Infinite)	0.995	0 (0-Infinite)	0.996	0 (0-Infinite)	0.996	0 (0-Infinite)	0.996	0 (0-Infinite)	0.996	0 (0-Infinite)	0.994
Dienogest	1.601 (1.006–2.547)	0.047	0.7 (0.367–1.339)	0.282	0.826 (0.253–2.70)	0.751	2.363 (0.723–7.719)	0.155	3.679 (1.126–12.018)	0.031	7.528 (4.254–13.32)	<0.001
Adjusted HR formula 2												
Age per 5 years	1.291 (1.086–1.535)	0.004	1.299 (1.04–1.623)	0.021	1.35 (1.047–1.741)	0.021	1.273 (0.988–1.639)	0.062	1.389 (1.074–1.798)	0.012	1.294 (1.046–1.601)	0.018
SES	0 (0-Infinite)	0.995	0 (0-Infinite)	0.996	0 (0-Infinite)	0.996	0 (0-Infinite)	0.996	0 (0-Infinite)	0.996	0 (0-Infinite)	0.994
Dienogest	1.599 (1.005–2.545)	0.048	0.698 (0.365–1.336)	0.278	0.816 (0.249–2.672)	0.736	2.419 (0.735–7.962)	0.146	3.826 (1.164–12.579)	0.027	8.436 (4.722–15.072)	<0.001
Number of GnRH agonist injections ^a^	1.062 (0.690–1.635)	0.785	0.968 (0.551–1.699)	0.909	1.094 (0.58–2.063)	0.782	0.943 (0.495–1.794)	0.857	0.872 (0.458–1.662)	0.678	0.629 (0.364–1.087)	0.097
CCI	1.064 (0.751–1.507)	0.728	0.918 (0.559–1.507)	0.735	0.95 (0.549–1.644)	0.855	0.717 (0.364–1.412)	0.335	0.715 (0.362–1.412)	0.334	0.95 (0.608–1.484)	0.823
Site of EMS ^b^	1.008 (0.739–1.374)	0.961	0.923 (0.607–1.405)	0.71	0.83 (0.498–1.382)	0.473	0.763 (0.446–1.303)	0.321	0.754 (0.44–1.292)	0.304	0.687 (0.439–1.076)	0.101

CCI, Charlson comorbidity index; CI, confidence interval; GnRH, gonadotropin-releasing hormone; HIRA, The Health Insurance Review and Assessment Service; HR, hazard ratio; SES, socioeconomic status. ^a^ The category of this variable is as shown in Table 2. ^b^ The category of this variable is as shown in Table 3.

## Data Availability

Due to the HIRA’s privacy policy, datasets generated during the present study are not publicly available. Our investigators have access to the dataset only during analysis and can achieve analysis results (tables, figures) without the raw data. If other investigators wish to access the raw data, they must be reviewed by HIRA.
